# Exploring perspectives on the benefits of a tuberculosis short-treatment regimen: a cross-sectional study on treatment experiences and perceptions

**DOI:** 10.36416/1806-3756/e20250059

**Published:** 2025-05-21

**Authors:** Fernando Pereira da Silva, João Pedro Ramos, Pedro Barbosa, Mariana Vieira, Raquel Duarte

**Affiliations:** 1. Departamento de Pneumologia, Unidade Local de Saúde da Guarda, Guarda, Portugal.; 2, EPIUnit ITR, Instituto de Saúde Pública da Universidade do Porto, Porto, Portugal.; 3. Instituto de Ciências Biomédicas Abel Salazar, Universidade do Porto, Porto, Portugal.; 4. Instituto Nacional de Saúde Doutor Ricardo Jorge - INSA-Porto - Porto, Portugal.

## TO THE EDITOR:

Tuberculosis is a transmissible disease and a leading cause of global mortality.^1^ Without proper treatment, the mortality rate can reach up to 50%,^1^ ^,^ ^2^ and is even higher in cases of poor adherence, infection with resistant strains, and immunosuppression.^3^ The standard treatment for drug-sensitive tuberculosis includes 2 months of isoniazid (H), rifampin (R), pyrazinamide (Z), and ethambutol (E), followed by 4-10 months of H and R, depending on clinical factors.^3^ ^,^ ^4^ However, adherence is also influenced by structural, personal, and social factors.^5^ The 4-month regimen incurs higher costs due to increased expenditure of rifapentine (Rpt) but requires fewer doses than the 6-month regimen. In contrast, the 6-month regimen involves longer follow-ups and more extensive directly observed therapy (DOT), raising costs for health care professionals (HCPs) and society.^6^ ^-^ ^8^ This study aimed to assess which tuberculosis treatment regimen, whether the gold standard or an alternative, was preferred by people with tuberculosis and by HCPs, as well as identifying the factors that most heavily influence their decision making.

**Figure 1:**
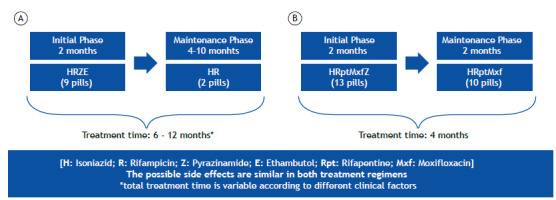
Diagram comparing the classic (A) and the alternative/shorter (B) treatment regimens.

In this cross-sectional study, HCPs and people with tuberculosis were asked to complete specific questionnaires between January and October of 2024. The HCP sample was intentionally selected, including pulmonology specialists, residents, and nurses registered with the *Sociedade Portuguesa de Pneumologia* and potentially interacting with tuberculosis patients in Pulmonology Diagnostic Centers (PDCs). That questionnaire was distributed online via the Society and senior professionals. A different questionnaire was administered to people with tuberculosis under treatment at the Vila Nova de Gaia PDC in Portugal, over the telephone or in person, using convenience sampling. Both questionnaires assessed the perceptions of participants regarding the two regimens (Figure 1), requiring them to choose from the perspective of a person with tuberculosis and to justify their reasoning. Participants reflected on treatment impact, challenges, duration, and medication intake burden. The HCP questionnaire additionally addressed financial and medication intake burden, treatment challenges, and the impact of DOT on mental health, social life, expenses, and treatment adherence. A descriptive statistical analysis was conducted using the IBM SPSS Statistics software package, version 29.0.1.0 (IBM Corporation, Armonk, NY, USA), alongside inductive and thematic analysis,^9^ to identify themes that could lead us to understand and describe the choice of the most appropriate/preferable treatment regimen. The study was approved by the Research Ethics Committee of the Local Health Care Unit of Vila Nova de Gaia/Espinho (CES 116/2024).

The HCP group comprised 41 participants. They were predominantly female (70.8%) with a mean age of 46.8 ± 14.8 years. Most were specialist physicians (63.4%), primarily pulmonologists (48.8%). Over half of these participants (56.1%) worked at a PDC during a mean period of 10.0 ± 9.4 years. The mean age of the patients with tuberculosis was 57.7 ± 14.7 years, with varying levels of education levels, half of whom having completed the 5^th^-6th school year. Only two of the patients were employed, and half reported a household income between €2,501 and €3,500 per month (Table 1).

**Table 1:** Sociodemographic characteristics.

Health Care Professionals (n = 41)		
Characteristic		Participants
Sex, n (%)	Male	12 (29.27%)
	Female	29 (70.75%)
Age, years	Mean [min-max]	46.80 [25-73]
Professional class, n (%)	Nurse	10
	Specialist Physician	26
	Medical Resident	5
Currently working at a PDC, n (%)	Yes	23 (56.10%)
	No	18 (43.90%)
Time working at a PDC, years	Mean [min-max]	10.0 [1-33]
Frequency of tuberculosis treatment prescription	1: Never	13 (31.7%)
	2: Rarely	6 (14.6%)
	3: Occasionally	8 (19.5%)
	4: Often	5 (12.2%)
	5: Very often	9 (22.0%)
Patients (n = 6)		
Characteristic		Participants
Sex, n (%)	Male	2
	Female	4
Age, years	Mean [min-max]	57,7 [36-81]
Tuberculosis involvement	Lung	4 (66.7%)
	Lymph nodes	1 (16.7%)
	Osteoarticular	1 (16.7%)
Expected treatment duration, months	6	5 (83.3%)
	12	1 (16.7%)
Current treatment phase	Initial	4 (66.7%)
	Maintenance	2 (33.3%)
Level of Education	5th to 6th grade	3 (50.0%)
	7th to 9th grade	1 (16.7%)
	High School	2 (33.3%)
Number of household members	1	2 (33.3%)
	2	1 (16.7%)
	3	2 (33.3%)
	4	1 (16.7%)
Professional situation	Employed	2 (33.3%)
	Unemployed	2 (33.3%)
	Retired	2 (33.3%)
Average household monthly income	Up to 500€	2 (33.3%)
	1501€ - 2500€	1 (16.7%)
	2501€ - 3500€	3 (50.0%)

PCD: Pulmonology Diagnostic Center.

A significant knowledge gap among HCPs regarding the shorter tuberculosis treatment regimen was noted; 56.1% of the HCPs were unaware of the alternative, mentioning their “unaware[ness] of the real effectiveness of the second [short] treatment and the scientific basis,” as well as their “lack of familiarity and limited experience” with it. However, 63.4% indicated they would choose to prescribe it, due to a “shorter treatment time.” Most HCPs (80.5%) believed people with tuberculosis would prefer a shorter regimen, as “patients want to resolve the situation as quickly as possible,” leading to “less psychological distress,” as prolonged treatment time was described as “one of the biggest complaints from users.”

Most people with tuberculosis preferred the shorter regimen due to the “shorter time of treatment” allowing them to “go back to normal life sooner; being with people and living my life without using a mask and having people stare at me.” They also valued “shorter time taking pills, going to consultations, and having to tell people what I have.” A person with tuberculosis mentioned, “It’s hard to take so many pills; I don’t like the amount I’m taking now, but then they taper off. [...] I’m living with my daughter, and she organizes my medication and gives it to me, so it wouldn’t be easy for her.” Additional considerations included the number of daily pills and potential side effects, as “patients usually get scared with the number of daily pills.”

Mental health was predominantly perceived as negative (48.8%) or neutral (29.3%), with “social stigma” and “prolonged isolation” cited as key concerns. However, one participant noted that treatment could “have a positive impact on mental health when the patient starts feeling better.”

Regarding financial burden, 39.0% of respondents rated treatment impact as “very negative” or “negative,” citing logistical challenges such as “the need for frequent trips to appointments”, which makes it more difficult by limited public transportation, and “the mandatory DOT” with “inflexible opening hours of health institutions.” as the most common issues. HCPs acknowledged these challenges, emphasizing that “emotional balance and social support are often key to successful treatment adherence,” and that “patients with financial issues, such as inability to work or lack of financial resources for basic needs, must be supported.” One respondent noted that “the sick leave is paid 100%,” while others reported, “remuneration during sick leave is lower than the wage.” These responses highlight the complexity of assessing the financial burden of tuberculosis treatment. Proposed solutions included “payment of travel expenses” and creating a “transport network that reduces patients’ travel costs.”

Tuberculosis treatment adherence was linked to personal, treatment-related, health care system-related, and social factors. Literacy about tuberculosis emerged as a key determinant, with professionals stressing the importance of “understanding the importance of medication, its purpose, duration, and side effects.” They emphasized the need to “inform and educate at the time of diagnosis and during treatment” to enhance “motivation for treatment” and adherence. However, many highlighted “the prolonged time [of treatment] and number of pills” and “side effects” as major issues. One person noted that “in the beginning, it felt like I was weak and sick.” 

Prolonged treatment, a high number of pills, and the “need to be ‘watched’ daily” affected adherence, calling for “shorter treatments and better-tolerated regimens” with “differentiated and adapted monitoring.” People with tuberculosis consistently rated treatment duration as “important” or “very important,” although opinions on the number of daily pills varied from “very Important” to “neutral.” Social support, particularly from family and the broader social context, was deemed crucial, with “poor social/family support, financial need, precarious work, (...) and chemical dependency” cited as major challenges.

This study examines the perspectives of HCPs on tuberculosis treatment preferences, comparing them with those of people with tuberculosis. A strong preference for shorter treatment regimens emerged, driven by reduced treatment duration, fewer health care visits, and psychological benefits. However, many HCPs were unaware of this option, revealing a knowledge gap. While shorter regimens may improve adherence, the higher pill burden remains a concern. Findings are limited to a single unit in one region and may not be fully generalizable. However, replicable methodologies can guide similar studies. Continued research, education, and tailored support are essential to improving adherence and treatment outcomes.^10^ Shorter regimens seem to be a promising step forward, but continued research is needed to evaluate their acceptability and improve health literacy and support, ultimately improving adherence and treatment outcomes.
